# Ovarian carcinoma in situ of presumable fallopian tube origin in a patient with Lynch syndrome: A case report^☆^

**DOI:** 10.1016/j.gynor.2013.05.002

**Published:** 2013-05-15

**Authors:** Kayo Inoue, Hiroshi Tsubamoto, Hiroyuki Hao, Kazuo Tamura, Tomoko Hashimoto-Tamaoki

**Affiliations:** a Department of Obstetrics and Gynecology, Hyogo College of Medicine, Nishinomiya, Japan; b Department of Obstetrics and Gynecology, Meiwa General Hospital, Nishinomiya, Japan; c Department of Surgical Pathology, Hyogo College of Medicine, Nishinomiya, Japan; d Department of Surgery, Hyogo College of Medicine, Nishinomiya, Japan; e Department of Life Science, Faculty of Science and Engineering, Kinki University, Higashiosaka, Japan; f Department of Genetics, Hyogo College of Medicine, Nishinomiya, Japan

**Keywords:** Lynch syndrome, Ovarian cancer, Carcinoma in situ

## Abstract

• Occult ovarian carcinoma of presumable fallopian tube origin in Lynch syndrome
• Atypical endometrial hyperplasia during a 10-year follow-up period after colon cancer
• Synchronous ovarian serous carcinoma in situ and borderline tumor in Lynch syndrome

## Introduction

Tubal intraepithelial carcinoma (TIC) has been proposed to be a precursor lesion of ovarian carcinoma based on studies of risk-reducing salpingo-oophorectomy (RRSO) specimens from hereditary breast and ovarian cancer patients [1]. On the other hand, one case of TIC has been reported in a patient with Lynch syndrome (LS) [2]. Furthermore, atypical proliferative (borderline) serous tumors account for only 4% of ovarian carcinomas in patients with LS. Detection of early-stage ovarian and fallopian tube cancers is difficult even with close follow-up of high-risk patients with LS [2–5]. This has complicated the identification of ovarian carcinomas of fallopian tube origin in patients with LS. Herein, we present a case of ovarian carcinoma in situ that presumably originated from the fallopian tube in a LS patient.

## Case

**Fig. 1:**
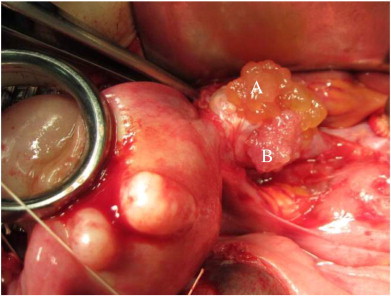
Gross appearance of tumors A and B at laparotomy. Tumor A was composed of yellowish exophytic nodular excrescences, and the adjacent tumor B showed white exophytic papillary excrescences.

**Fig. 2:**
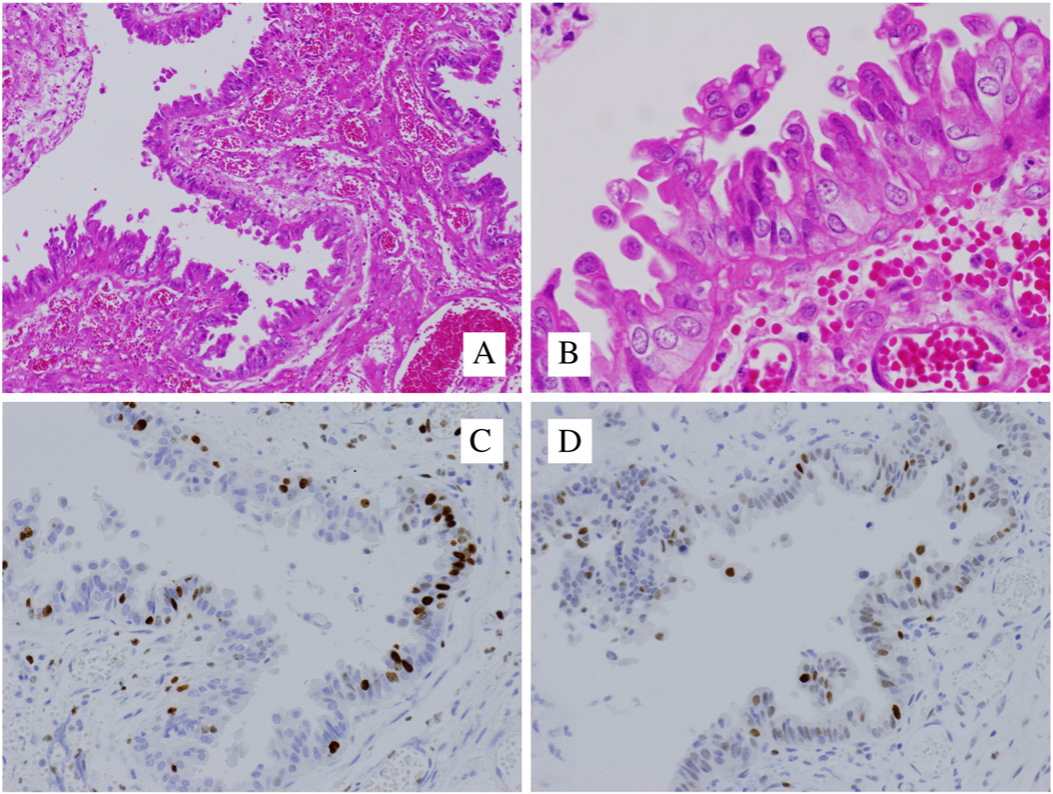
Tumor A. Hematoxylin and eosin (H&E) stain showing exophytic papillary growth composed mostly of a flat proliferation of cells (panel A; × 200) and mild unclear atypia of the lesional cells (panel B; × 400). Immunohistochemical staining of Ki-67 expression. The Ki-67 proliferation index of the tumor cells was 20% (panel C; × 200). Immunohistochemical staining showing the patchy expression of p53 (panel D; × 200).

**Fig. 3:**
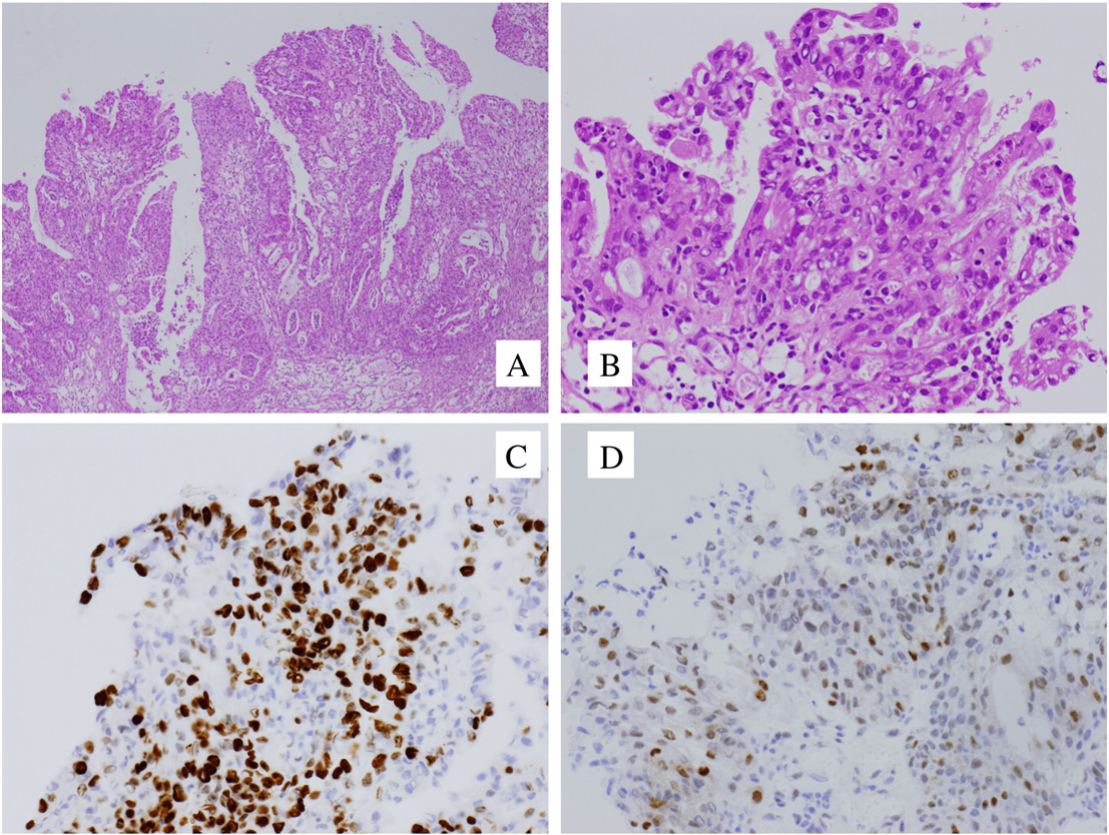
Tumor B. H&E stain showing complex papillary growth consisting of stratified atypical tumor cells (panel A; × 40) and exophytic growth of heterogeneous tumor cells with high-grade nuclear atypia, stratification, loss of polarity, and absence of stromal invasion (panel B; × 200). Immunohistochemical staining of Ki-67 expression. The Ki-67 proliferation index of the tumor cells was 55% (panel C; × 200). Immunohistochemical staining showing the patchy expression of p53 (panel D; × 200).

A 34-year-old multipara woman was diagnosed with sigmoid colon cancer and underwent total colectomy. Her family history matched the Amsterdam criteria for LS. Genetic testing showed the presence of a pathogenic MLH1 mutation (Exon3: IVS3 + 1 g>a). The patient was referred to a gynecologist and subsequently underwent semiannual surveillance by transvaginal ultrasonography and endometrial cytology. After 10 years, an endometrial biopsy revealed complex atypical hyperplasia. Adnexal masses were not detected upon transvaginal ultrasonography and magnetic resonance imaging, and the carbohydrate antigen-125 level was 15 U/mL (normal, < 35 U/mL). Although the advantages of total abdominal hysterectomy (TAH) with preventive bilateral salpingo-oophorectomy (BSO) were explained and this approach was recommended by cancer genetics professionals, the patient strongly desired preservation of the ovaries in the absence of an intraoperative malignancy diagnosis. Laparotomy revealed the adherence of the right fallopian tube to the right ovary and the presence of exophytic papillary excrescences on the surface of the right ovary (tumors A and B in Fig. 1). Tumor A was composed of yellowish small nodules, whereas the adjacent tumor B showed white fine papillary structures. Tumor B was resected for intraoperative pathologic evaluation and was diagnosed as being malignant. TAH/BSO was performed, and the hysterectomy specimen indicated complex atypical endometrial hyperplasia of the uterus. Histopathology revealed tumor A to be an atypical proliferative (borderline) serous tumor with tubal type epithelium containing a mixture of ciliated and secretory cells (Fig. 2, panels A and B). Histopathology of tumor B, which was adjacent to tumor A, revealed a high-grade serous carcinoma in situ. Tumor B showed tubal type epithelium with a papillary growth pattern that was composed of stratified secretory cells with high-grade nuclear atypia. Neither basement membrane destruction nor invasion of the underlying stroma was detected. Therefore, serous carcinoma in situ was our final diagnosis, which was confirmed by intradepartmental reviews and extradepartmental consultations (Fig. 3, panels A and B). Tumor B was resected for intraoperative diagnosis, and paraffin blocks of tumor A were prepared from the TAH/BSO specimens. Thus, the border of tumors A and B was not available for microscopic observation. Immunohistochemical analysis of tumor A showed patchy p53 positive staining and a Ki-67 proliferative index of 20% (Fig. 2, panels C and D). Tumor B stained more diffusely and strongly for p53 and Ki-67 (proliferative index, 55%) than tumor A (Fig. 3, panels C and D). The fimbriated end of the right fallopian tube was unclear because of adhesion to the ovary. Atypia was not present in the fallopian tube segments. Pelvic washing was positive. The patient did not wish to receive adjuvant chemotherapy. She is currently alive without evidence of disease 22 months after TAH/BSO.

## Discussion

High-grade serous ovarian carcinoma has been presumed to arise from TIC. Detailed pathological studies of RRSO specimens from women with inherited *BRCA* mutations have shown the presence of TIC, and a precursor of TIC has been found in neighboring tubal cells that are morphologically normal but immunohistochemically positive for p53, designated as a p53 signature [6]. Low-grade serous ovarian cancers could also be derived from the tubal epithelium [7], and in rare cases, high-grade ovarian carcinomas may arise from serous borderline tumors [8,9].

To our knowledge, a serous borderline tumor that coexists with high-grade serous carcinoma in situ on the ovarian surface and p53 signatures in patients with LS have not been reported. Dehari et al. reported 6 cases of sporadic high-grade serous ovarian carcinomas that presumably progressed from borderline or low-grade tumors [9]. p53 mutations were not detected in these 6 cases. In contrast, in our case, the borderline serous tumor (tumor A), even in its earliest stage of development, and the high-grade serous carcinoma in situ (tumor B) showed overexpression of p53. This suggests the existence of a tumorigenic pathway in which p53 mutations give rise to borderline ovarian tumors that further progress to high-grade carcinomas in patients with LS. However, adhesions between the right fimbria and ovary from the previous colon surgery may have caused ovarian tumors to originate from the tubal epithelium in this LS patient.

TIC lesions with positive washing have been reported in *BRCA* mutation carriers. However, diagnosis of serous ovarian carcinoma in situ as the only lesion with positive washing is very rare in patients with LS.

## Conclusion

We reported a rare case of ovarian carcinoma in situ, with a p53 signature, originating from the fallopian tube epithelium and the coexistence of serous ovarian carcinoma in situ and serous borderline tumor in a LS patient.

## Conflict of interest statement

The authors have no financial conflicts of interest to disclose.
